# Deep learning–based automatic segmentation of MRONJ lesions on CBCT images

**DOI:** 10.1186/s12903-026-08022-1

**Published:** 2026-03-05

**Authors:** Alp Pınarbaşı, Meryem Kaygısız Yiğit, Meryem Etöz

**Affiliations:** Department of Oral and Maxillofacial Radiology, Faculty of Dentistry, Erciyes University, Melikgazi, Kayseri, 38039 Turkey

**Keywords:** Artificial intelligence, Deep learning, nnU-Net v2, Osteonecrosis

## Abstract

**Background:**

Medication-related osteonecrosis of the jaw (MRONJ) is a serious pathology that can cause bone necrosis in the jaws of patients using antiresorptive or antiangiogenic drugs, often triggered by conditions such as dental infections, periodontal diseases, surgical procedures, or trauma. The purpose of this study was to evaluate the feasibility and segmentation performance of an nnU-Net v2–based deep learning model for the automatic segmentation and voxel-level localization of MRONJ lesions on cone-beam computed tomography (CBCT) images.

**Methods:**

In this study, cone beam computed tomography images of 52 patients with MRONJ were used. All images were manually annotated by experienced oral and maxillofacial radiologists using 3D Slicer software to generate ground truth segmentations. The nnU-Net v2 3D low-resolution architecture, implemented within an automated training pipeline, was trained for 1000 epochs.

Model evaluation was performed within the framework of the default five-fold cross-validation strategy of nnU-Net v2. Model performance was evaluated using accuracy, recall (sensitivity), precision, Dice coefficient (DC), Intersection over Union (IoU) and 95% Hausdorff distance (95% HD) metrics.

**Results:**

The nnU-Net v2 algorithm generated predictions on the evaluated cases of MRONJ patients. The algorithm achieved average accuracy, recall, and precision scores of 0.999, 0.707, and 0.743, respectively. In addition, the DC, 95% HD, and IoU values were 0.716, 4.045 mm, and 0.569, respectively.

**Conclusions:**

This study demonstrates the feasibility of nnU-Net v2–based automatic MRONJ segmentation on CBCT images. Although limited by dataset size, the results suggest potential clinical utility as a supportive tool rather than a standalone diagnostic system.

## Background

Medication-related osteonecrosis of the jaw (MRONJ) represents a severe adverse event associated with the administration of antiresorptive or antiangiogenic agents. It is clinically defined as the persistence of exposed bone or intraoral/external fistulae lasting longer than eight weeks in patients without a history of craniofacial radiotherapy [1]. The risk of MRONJ depends on multiple factors, such as antiresorptive and/or antiangiogenic drug therapy, existing dental conditions, invasive procedures, advanced age, sex, anatomical characteristics, systemic comorbidities, and genetic predisposition [2].

One of the major challenges in managing MRONJ is early diagnosis and accurate staging. The early stages of MRONJ may resemble odontogenic infections or even remain asymptomatic, and the clinical presentation does not always reflect the true extent of the disease [3]. Diagnostic criteria based solely on clinical findings, without the support of imaging modalities, may prevent the recognition of “Stage 0 MRONJ,” potentially delaying treatment that could halt disease progression. Nearly half of patients classified as Stage 0 experience progression to more advanced stages with poorer prognosis. Approximately 30% of MRONJ patients present in Stage 0, and more than 60% of these individuals tend to demonstrate radiographic abnormalities [4]. Therefore, radiographic evaluation plays a critical role in assessing the extent of necrotic bone involvement and in guiding the appropriate surgical approach [3].

Cone-beam computed tomography (CBCT) is recommended for the evaluation of MRONJ due to its high spatial resolution, small voxel size, acceptable radiation dose, and limited field of view (FOV) that is well-suited for assessing the alveolar bone region [5]. Compared to panoramic radiography, CBCT offers superior performance in detecting sequestra, assessing bone quality, delineating lesion boundaries, and providing various other diagnostic advantages [6]. Torres et al. reported that CBCT is more effective than conventional computed tomography (CT) and magnetic resonance imaging (MRI) in evaluating cortical bone structures [7]. However, CBCT remains insufficient for the assessment of soft tissues [8].

In recent years, advances in machine learning (ML) and deep learning (DL) have enabled the automated analysis of complex medical imaging data, particularly in tasks such as detection, classification, and segmentation [9]. Unlike traditional ML approaches that rely on handcrafted features, DL models—especially convolutional neural networks (CNNs)—can automatically learn hierarchical feature representations directly from imaging data, making them particularly suitable for volumetric modalities such as CBCT [10].

In oral and maxillofacial radiology, artificial intelligence (AI) techniques have been increasingly applied not only for diagnostic purposes but also for automated image interpretation and quantitative analysis. AI has been applied to the diagnosis of dental caries, periodontal diseases, osteosclerosis, odontogenic cysts and tumors, as well as paranasal sinus and temporomandibular joint disorders. Beyond diagnosis, AI supports various tasks in dentistry, including image analysis, tooth segmentation and localization, assessment of bone quality (e.g., osteoporosis), bone age estimation using hand–wrist radiographs, and identification of cephalometric landmarks [11]. Convolutional neural networks (CNNs), a deep learning technique known for their strong image analysis capabilities, have been applied to both planar radiographs and volumetric CBCT data [11, 12].

Despite the growing body of literature on AI-based analysis of maxillofacial pathologies, studies specifically focusing on the automatic segmentation of MRONJ lesions on CBCT images remain limited. Given that MRONJ diagnosis relies on a combination of clinical findings and radiographic features, an AI-based segmentation approach has the potential to provide objective and reproducible support for radiologists and clinicians by facilitating standardized radiographic assessment and lesion delineation.

Therefore, the present study was designed as a preliminary feasibility investigation to assess the applicability of deep learning–based automatic MRONJ segmentation using CBCT data. Recent studies have highlighted the role of CBCT in the assessment of MRONJ, particularly for detecting early osseous changes and defining lesion extent [13, 14], while artificial intelligence–based approaches have increasingly been applied in dentomaxillofacial imaging. However, applications specifically targeting MRONJ remain limited. While lesion detection may indicate the presence of MRONJ, segmentation provides spatial delineation of lesion extent, which is essential for surgical planning, volumetric assessment, and longitudinal follow-up. Therefore, segmentation offers greater clinical utility than binary detection alone.

The aim of this research is to evaluate the performance of an nnU-Net v2–based CNN model for the automatic segmentation and voxel-level localization of MRONJ lesions on CBCT images with the intention of exploring its potential role as a supportive tool rather than a standalone diagnostic system.

## Materials and methods

### Study design

This retrospective study was carried out at the Department of Oral and Maxillofacial Radiology, Faculty of Dentistry, Erciyes University, adhering to the ethical principles of the Declaration of Helsinki for medical research. Approval was granted by the Erciyes University Health Sciences Research Ethics Committee (decision no: 2024/299). Due to the retrospective design of the study and the use of fully anonymized imaging data, the requirement for informed consent to participate was waived by the Erciyes University Health Sciences Research Ethics Committee, in accordance with national regulations. The study design and reporting followed the STROBE guidelines for observational studies, while the development and evaluation of the artificial intelligence–based model adhered to the Checklist for Artificial Intelligence in Medical Imaging (CLAIM).

### Preparation of the dataset

Between 2015 and 2024, CBCT images of 63 patients with a diagnosis of MRONJ were reviewed at the Department of Oral and Maxillofacial Radiology, Faculty of Dentistry, Erciyes University. Eleven images deemed diagnostically inadequate were removed from the study. A total of 52 patients with clinically and pathologically confirmed MRONJ, without any other jaw pathology and possessing diagnostically sufficient CBCT images, were included in the study. Ages of the patients varied from 47 to 83 years and no gender distinction was made.

### Imaging parameters

CBCT scans were obtained using a NewTom 5G CBCT system (FP, Quantitative Radiology, Verona, Italy). Scanning parameters included 110 kV, 3–5 mA, a voxel size of 0.25 mm, and a field of view (FOV) of 18 × 16, 15 × 12, or 12 × 8 cm², with a slice thickness of 0.25 mm and an exposure time of 5.4 s. The acquired images were then exported in DICOM (Digital Imaging and Communications in Medicine) format using NNT software (version 3.0; NewTom, Verona, Italy).

### Ground truth and labeling

Ground truth refers to reference data with known accuracy that is used for training AI models and evaluating their performance [15]. Data labeled by experts and used as a reference constitute the ground truth.

**Fig. 1:**
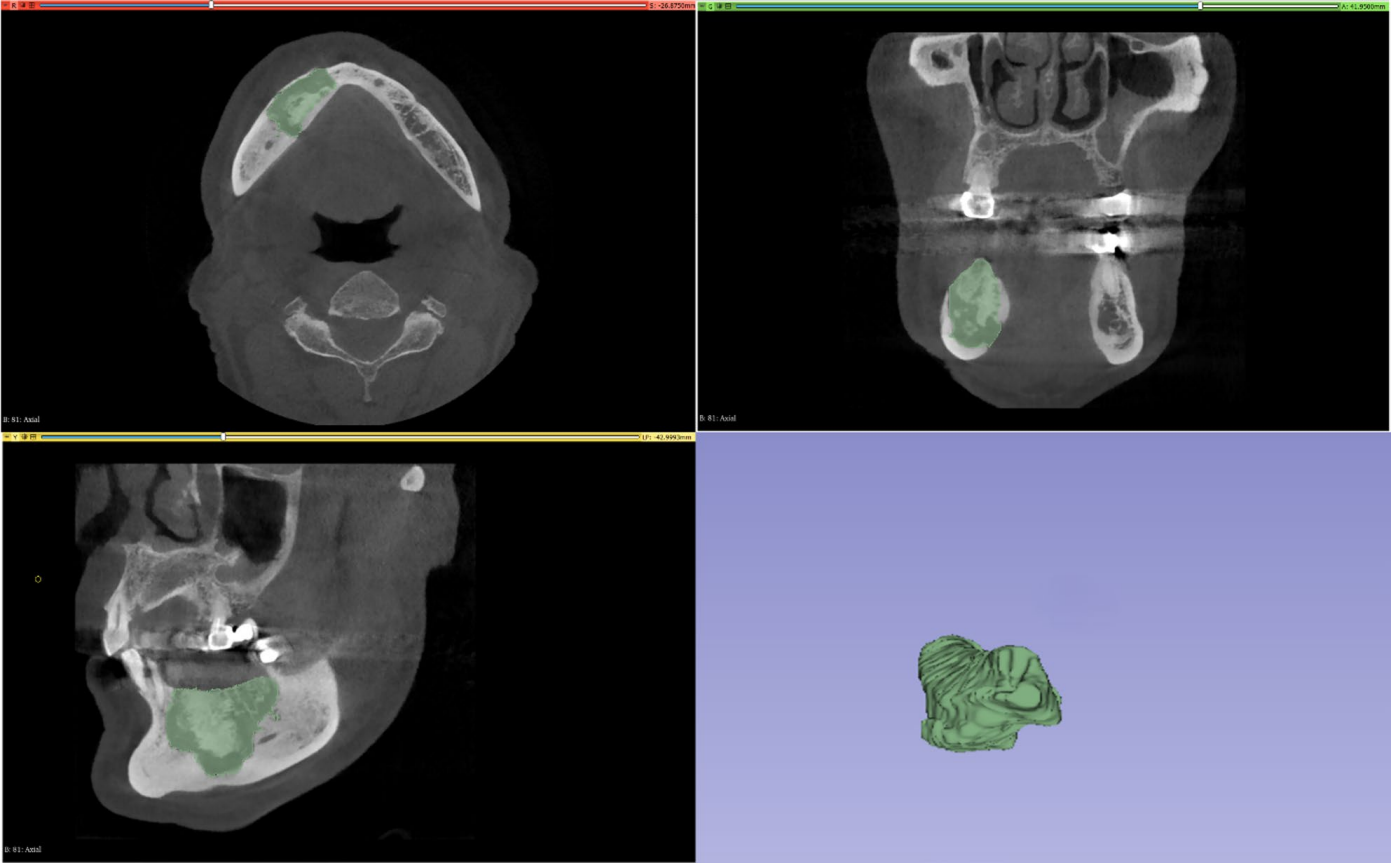
Labelling on axial, coronal and sagittal CBCT images and 3D model

CBCT images exported in DICOM format were annotated using 3D Slicer 5.4.0 (http://www.slicer.org). Two oral and maxillofacial radiology researchers, with four and three years of experience respectively, manually annotated the images using a polygon-based method on sagittal, coronal, and axial planes (Fig. 1). A radiologist specialized in oral and maxillofacial imaging, with thirteen years of expertise, examined all annotated datasets. Discrepancies between annotators were resolved by consensus under the supervision of the senior radiologist. The fully annotated datasets were exported in Neuroimaging Informatics Technology Initiative (NIfTI) format.

### Model training

**Fig. 2:**
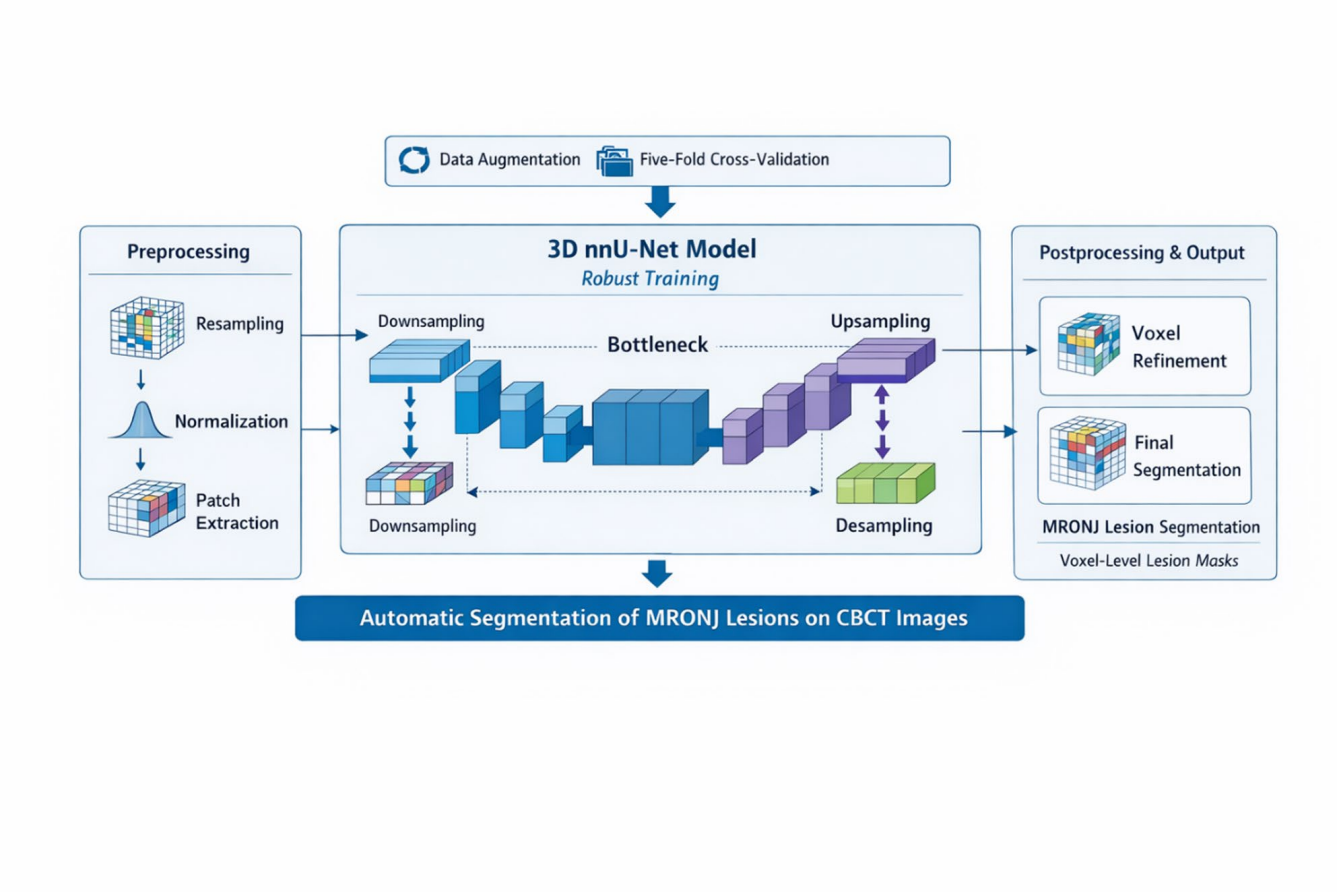
Illustration of the nnU-Net v2 architecture and training workflow applied for automatic MRONJ lesion segmentation. The diagram shows the encoder–decoder structure and voxel-level segmentation output within the self-configuring nnU-Net v2 framework

The nnU-Net v2 framework was employed in its self-configuring mode, in which architectural design, patch size, batch size, learning rate schedule, and data augmentation strategies are automatically determined based on the properties of the input dataset. Model training was performed using the default nnU-Net v2 settings, including stochastic gradient descent optimization and a compound Dice–cross-entropy loss function. The model was trained by CranioCatch (CranioCatch, Eskişehir, Turkey). The nnU-Net v2 3D low-resolution configuration, developed using Python (v3.6.1; Python Software Foundation, Wilmington, DE, USA) with the PyTorch library, was employed for training (Fig. 2). Using the CBCT datasets, the model was trained for 1000 epochs.

The nnU-Net v2 framework utilizes a default five-fold cross-validation strategy, in which the dataset is automatically divided into five folds for training and validation to enhance robustness and reduce overfitting. The dataset consisted of 52 CBCT volumes, and each fold was iteratively used as an independent validation (unseen) set while the remaining four folds were utilized for model training. This strategy was adopted to maximize data utilization and to enable robust performance evaluation across all cases, which is a commonly used and accepted validation approach in deep learning studies involving limited sample sizes where an independent external test set is not feasible. In accordance with the nnU-Net pipeline, model performance was evaluated across the validation folds rather than using a fixed hold-out test set.

**Fig. 3:**
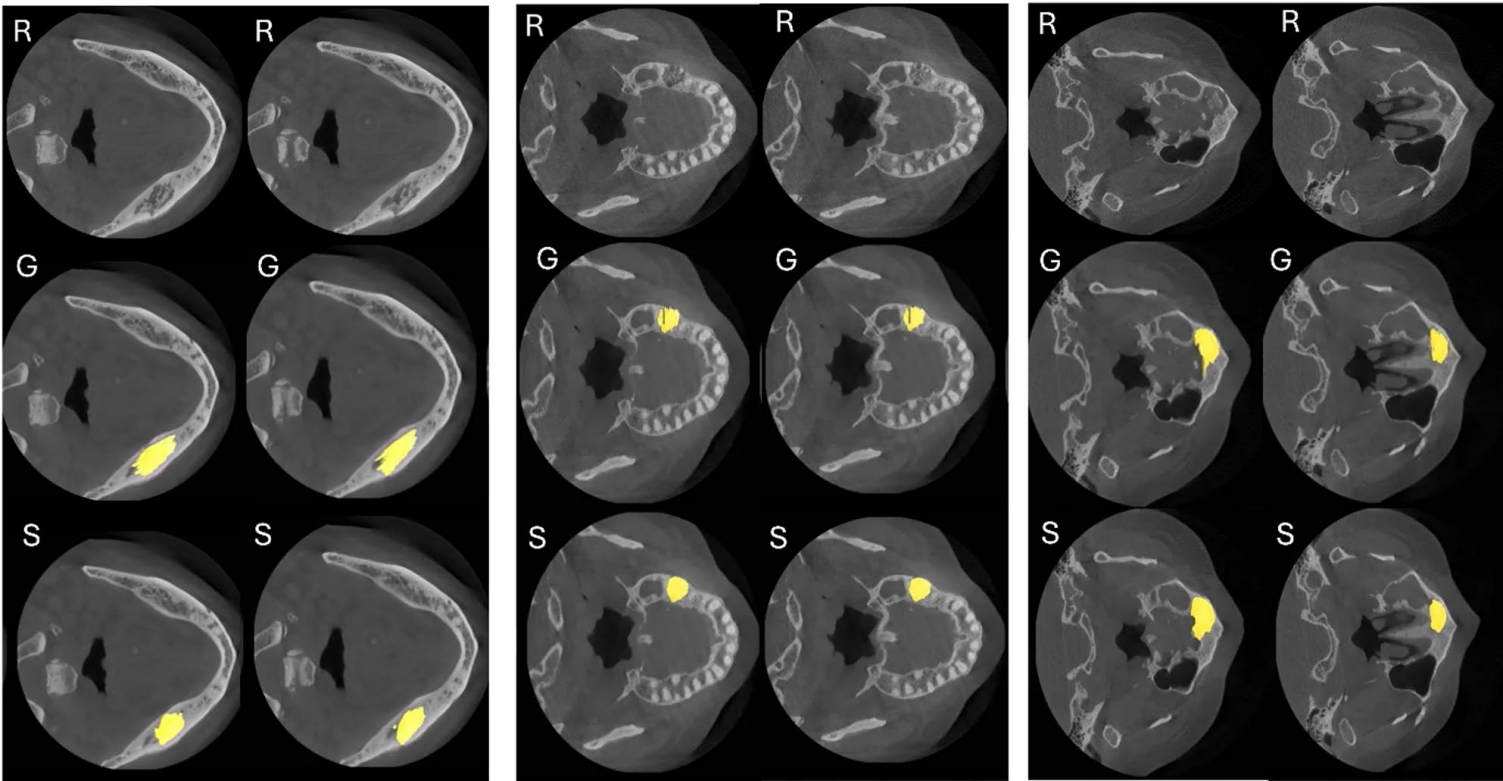
Representative axial CBCT images from three different patients demonstrating nnU-Net v2–based volumetric segmentation of MRONJ lesions. For each patient, two different axial slices are presented to illustrate lesion extent at different levels within the same CBCT volume. R: Raw, G: Ground Truth, S: Segmentation output

Model predictions were generated in a volumetric manner, with results visualized on axial CBCT slices for illustrative purposes (Fig. 3).

### Evaluation metrics for model performance

The performance of the model was evaluated using the accuracy, recall (sensitivity), precision, Dice coefficient (DC), Intersection over Union (IoU), 95% Hausdorff distance (95% HD) and area under the curve (AUC). Instead of relying on a fixed overlap threshold, quantitative overlap- and distance-based metrics were used to provide a more comprehensive assessment of segmentation performance. True positive (TP), false positive (FP), and false negative (FN) values were calculated at the voxel-level based on 3D segmentation masks. Performance metrics were first computed separately for each CBCT volume within the five-fold cross-validation framework and subsequently averaged across all validation folds (Table 1).

**Table 1:** Metrics used for evaluating model performance

Metric	Definition	Formula
True Positive (TP)	Number of voxels correctly predicted as MRONJ lesion voxels and also labeled as lesion in the ground truth segmentation.	–
False Positive (FP)	Number of voxels incorrectly predicted as MRONJ lesion voxels while labeled as non-lesion voxels in the ground truth.	–
False Negative (FN)	Number of voxels labeled as MRONJ lesion voxels in the ground truth but incorrectly predicted as non-lesion voxels by the model.	–
Accuracy	Proportion of correctly classified voxels among all voxels in the 3D CBCT volume.	$\frac{TP}{TP+FP+FN}$
Recall (Sensitivity)	Ability of the algorithm to correctly detect actual positives.	$\frac{TP}{TP+FN}$
Precision	Proportion of predicted positives that are truly positive.	$\frac{TP}{TP+FP}$
Dice Coefficient (DC)	Measures overlap between predicted segmentation (A) and ground truth (B). Value ranges from 0 (no overlap) to 1 (perfect overlap).	$\frac{2\mathrm\mid{A}\cap B\mathrm\mid}{\mathrm\mid{A\mid}+\mid B\mathrm \mid}or\frac{2TP}{2TP+FP+FN}$
95% Hausdorff Distance (95% HD)	Measures distance between prediction and ground truth shapes. Less sensitive to outliers compared to standard Hausdorff distance. Lower is better.	${\mathrm d}_{\mathrm H95}\left(\mathrm A,\mathrm B\right)=\max\left({\mathrm d}_{95}\left(\mathrm A,\mathrm B\right),\;{\mathrm d}_{95}\left(\mathrm B,\mathrm A\right)\right)$
Intersection over Union (IoU)	Measures overlap between prediction (A) and ground truth (B). Equivalent to Jaccard Index.	$\frac{\mathrm\mid{A}\cap B\mathrm \mid}{\mathrm\mid{A}\cup B\mathrm \mid}$
ROC Curve & AUC	ROC curve shows performance across thresholds. AUC summarizes classification performance (1 = perfect, 0.5 = random, < 0.5 = poor).	–

∩ Intersection, ∪ Union

## Results

The nnU-Net v2 3D low-resolution algorithm performed automatic segmentation of MRONJ lesions on CBCT images. Across the evaluated cases, the algorithm achieved mean accuracy, precision, and recall values of 0.999, 0.743, and 0.707, respectively. Segmentation-specific performance metrics yielded a mean Dice coefficient (DC) of 0.716, a mean 95% Hausdorff distance (95% HD) of 4.045 mm, and a mean Intersection over Union (IoU) of 0.569. Performance metrics are reported as mean ± standard deviation, calculated across the individual validation samples evaluated within the five-fold cross-validation framework (Table 2). The highest and lowest values obtained for the evaluated cases were as follows: DC, 0.857–0.522; IoU, 0.75–0.353; precision, 0.934–0.467; and recall, 0.908–0.592.

**Table 2:** Segmentation results of the nnU-Net v2 algorithm

METRICS	RESULTS Mean ± SD
True Positive (TP)	143,430 voxels
False Positive (FP)	30,452 voxels
False Negative (FN)	50,018 voxels
Accuracy	0.999 ± 0.001
Recall (Sensitivity)	0.707 ± 0.122
Precision	0.743 ± 0.179
Dice Coefficient (DC)	0.716 ± 0.125
95% Hausdorff Distance (mm)	4.045 ± 2.50
Intersection over Union (IoU)	0.569 ± 0.147

TP, FP, and FN denote voxel-level counts aggregated across all validation folds

*SD* Standard Deviation

**Fig. 4:**
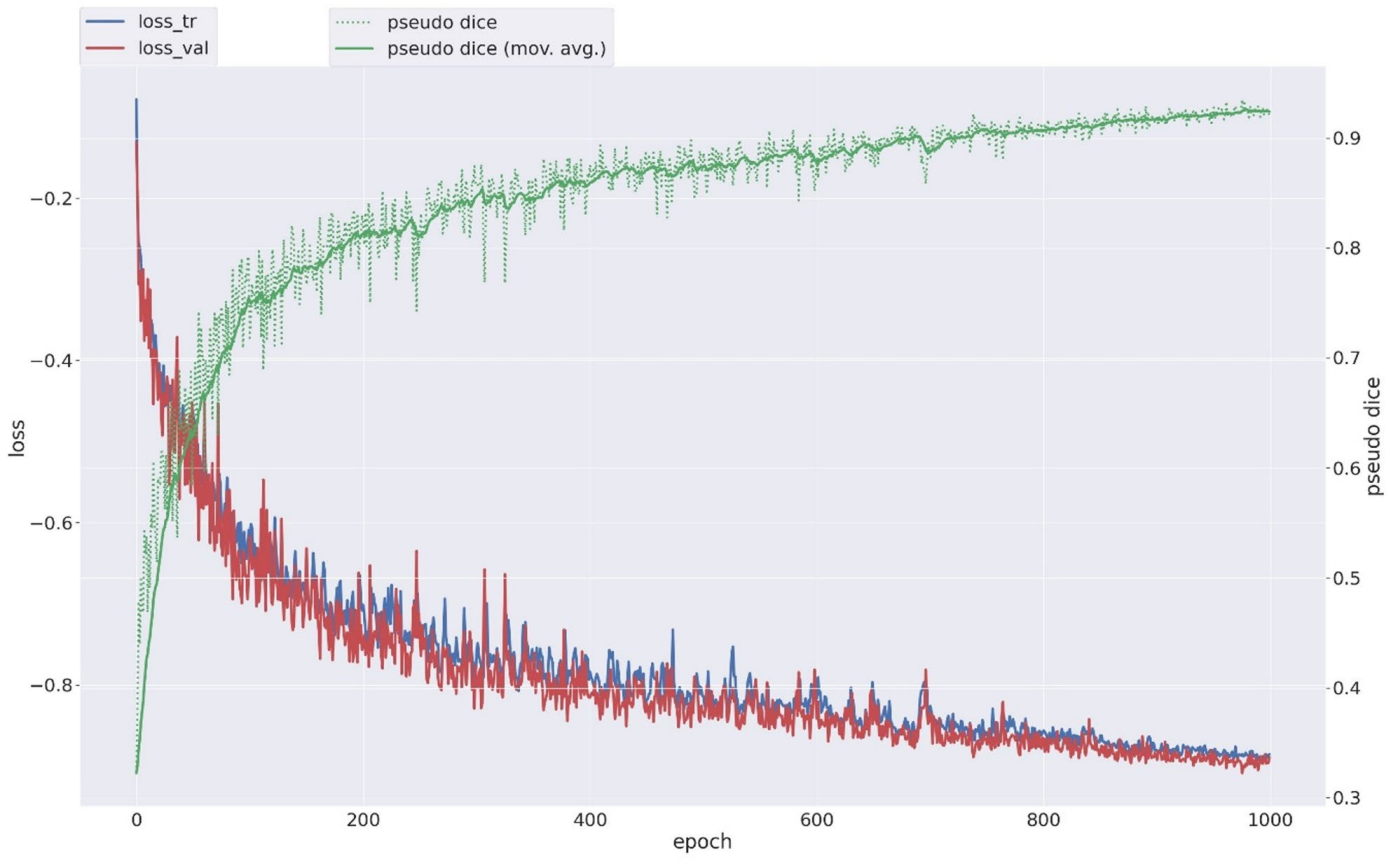
Changes in loss functions and pseudo DC during 1000 epochs of data training

As shown in Fig. 4, during the training process, both the training and validation losses decreased, while the pseudo DC steadily increased, indicating stable convergence of the training process and consistent learning behavior.

**Fig. 5:**
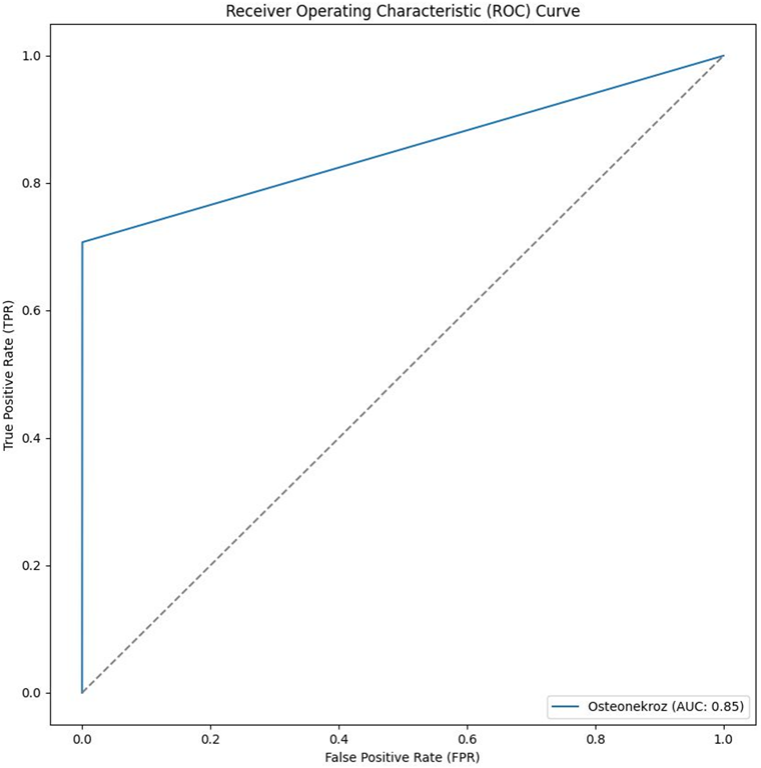
ROC curve and AUC

The ROC curve illustrating the voxel-level segmentation performance of the model in MRONJ lesions is shown in Fig. 5. The AUC value was calculated as 0.85 and is reported as a supportive performance indicator. The ROC curve and AUC were calculated at the voxel level and should be interpreted as indicators of segmentation performance within MRONJ lesions rather than diagnostic discrimination at the patient level.

## Discussion

With the rapid advancement of deep learning technologies, AI has seen increasing adoption across many disciplines. Deep learning algorithms have effectively tackled numerous clinical challenges in medicine and have created new opportunities for dental applications. In dentomaxillofacial radiology, AI is particularly utilized to aid in diagnosis, support treatment planning, and predict specific treatment outcomes [16]. Beyond diagnostic accuracy, AI has also gained attention for its potential to improve workflow efficiency and standardize image interpretation, particularly in imaging-intensive disciplines such as oral and maxillofacial radiology. Its ability to save time while offering reliable diagnostic support to dentists of varying experience levels makes it a vital tool in the field.

In dentistry, CBCT is widely utilized for diagnostic purposes, treatment planning, and surgical interventions because it offers comprehensive three-dimensional data regarding the teeth and adjacent alveolar bone, offering advantages over other imaging techniques [17]. Nevertheless, clinicians with less experience have noted poor inter- and intra-observer consistency when evaluating CBCT scans [18]. Consequently, incorporating AI into CBCT, which is routinely used in dental practice, can streamline and speed up examinations, reduce clinician workload, and allow for more objective and reproducible assessments in a shorter time [16].

Recent advances in AI have enabled its integration into CBCT imaging for a wide range of dental and maxillofacial applications. AI algorithms are utilized in areas such as osteoporosis diagnosis [19], cephalometric landmark detection [20, 21], identification of periapical diseases [22, 23], and caries detection [24]. By facilitating the recognition of the second mesiobuccal canal [25] and vertical root fractures [26], AI improves diagnostic accuracy in endodontic procedures. It further supports preoperative implant planning by evaluating alveolar bone volume [27] and localizing the mandibular canal [28], thereby contributing to safer and more precise implant placement. In addition, AI assists in the assessment of temporomandibular joint disorders [29]. Its applications also include automatic tooth segmentation [30] and the detection of supernumerary teeth such as mesiodens [31]. Collectively, these studies demonstrate that AI applications in dentistry are primarily intended to support clinicians by enhancing efficiency and consistency, rather than serving as standalone diagnostic systems.

In recent years, numerous image processing and segmentation studies have been conducted in medicine and dentistry using nnU-Net–based models. While a significant portion of this research has focused on anatomical structures, there are also studies specifically addressing pathological lesions.

In the literature, studies [32, 33] involving the segmentation of MRONJ lesions have primarily utilized segmentation to measure lesion volumes. Gürses et al. [34], on the other hand, labeled regions of sclerotic bone, necrotic bone, and radiographically normal bone in the mandibles of 7 patients with BRONJ and 8 healthy controls using axial CBCT images, and evaluated the performance of a support vector machine–based algorithm in detecting BRONJ in the mandible by comparing diseased and healthy groups. Remarkably high scores, exceeding 0.99 for accuracy, recall, precision, and specificity, were reported. As far as we are aware, our study is among the first in the literature to employ an nnU-Net v2–based deep learning algorithm for the segmentation and voxel-level localization of MRONJ lesions using CBCT images.

Previous studies utilizing nnU-Net–based deep learning models with 3D CBCT imaging have consistently demonstrated high segmentation performance for well-defined anatomical structures, including the paranasal sinuses [35, 36], nasolacrimal canal [37], and mandibular canal [38]. These structures typically exhibit regular morphology and clearly delineated boundaries, which facilitate accurate automatic segmentation. However, several reports have also noted a decline in segmentation performance when nnU-Net–based models are applied to anatomically complex regions or structures with less distinct borders [39]. Collectively, these findings indicate that while nnU-Net v2 performs robustly in anatomical segmentation tasks, segmentation of pathological lesions—characterized by irregular shape, heterogeneous density, and ill-defined margins—remains inherently more challenging.

Compared to anatomical structures, segmentation of pathological jaw lesions using 3D CBCT imaging poses greater challenges due to heterogeneous density, irregular morphology, and ill-defined boundaries. Previous studies applying nnU-Net and other deep learning architectures to cystic and tumoral jaw lesions have reported consistently lower performance metrics than those achieved in anatomical segmentation tasks, while still demonstrating clinically meaningful results [40–43]. These studies indicate that deep learning models can effectively support lesion detection and segmentation, albeit with reduced accuracy compared to well-defined anatomical targets. Collectively, the literature highlights the intrinsic difficulty of pathological lesion segmentation on CBCT and underscores the need for further task-specific model development.

Beyond CBCT-based studies, nnU-Net–based segmentation approaches have also been applied to MRI and CT datasets across various pathological conditions. Prior research has demonstrated that while nnU-Net can achieve strong performance in structured challenges such as brain tumor segmentation [44], its accuracy tends to decrease when applied to poorly defined malignant lesions with heterogeneous appearance [45]. These findings suggest that segmentation performance is highly dependent on lesion characteristics and boundary definition rather than the imaging modality alone. Collectively, this body of literature reinforces the notion that automatic segmentation of complex pathological lesions remains inherently challenging, even when advanced deep learning architectures are employed.

In the present study, given the known variability in lesion size, shape, and location, the nnU-Net v2–based model demonstrated consistent performance in terms of recall, precision, Dice coefficient, and 95% Hausdorff distance. The relatively lower IoU values observed may be attributed to the complex morphology and irregular margins of MRONJ lesions, which pose inherent challenges for precise boundary delineation.

Radiographic evaluation plays a central role in the management of MRONJ, as clinical examination alone often underestimates the true extent of bone involvement. While clinical staging is primarily based on the presence of exposed bone, CBCT imaging enables a more comprehensive assessment by revealing non-specific osteosclerotic changes, cortical irregularities, and the precise localization of sequestra that may be imperceptible on two-dimensional radiographs [46, 47]. In particular, for patients in advanced disease stages—such as Stage 2, which constituted the majority of our study population—accurate delineation of necrotic bone margins is critical for effective surgical debridement and treatment planning [48].

The segmentation of MRONJ lesions is inherently challenging due to their complex radiological appearance, characterized by heterogeneous bone density and ill-defined boundaries. Unlike well-circumscribed anatomical structures, MRONJ lesions frequently present with diffuse osteosclerosis and irregular sequestrum formation [49]. These pathological characteristics likely contribute to the relatively lower IoU and Dice scores observed in our model compared with anatomical segmentation tasks. Nevertheless, achieving a Dice coefficient of 0.716 indicates that the proposed nnU-Net v2–based approach can meaningfully objectify these complex radiographic features. The transition from manual radiographic indices such as the composite radiographic index to automated three-dimensional segmentation may help reduce inter-observer variability and improve workflow efficiency in oral and maxillofacial radiology [49, 50].

This study has several limitations. First, the relatively limited number of MRONJ cases reflects the rarity of the condition and restricts the generalizability of the findings. Therefore, the results should be interpreted as a preliminary feasibility assessment rather than a definitive clinical validation. While the nnU-Net v2 framework incorporates internal five-fold cross-validation to reduce overfitting, future studies with larger, multi-center datasets are required to improve robustness and external validity. Although patients with pathological lesions other than MRONJ were excluded from the study, factors such as reductions in bone density due to systemic conditions or medications, extraction sockets, and radiolucent anatomical structures such as the mandibular canal, nasopalatine canal, and maxillary sinus may complicate the training process of the model. In addition, the dataset used in the present study consisted exclusively of patients with confirmed MRONJ and did not include healthy individuals or non-MRONJ jaw pathologies. Consequently, the proposed model is limited to lesion localization and segmentation within MRONJ-affected CBCT volumes and cannot be applied for patient-level discrimination between MRONJ and non-MRONJ cases. Accordingly, all reported performance metrics, including the ROC curve and AUC, reflect voxel-level segmentation performance of MRONJ-affected regions rather than diagnostic classification or patient-level detection. Future studies incorporating appropriate control groups are necessary to enable true diagnostic differentiation.

## Conclusions

This study presents a preliminary feasibility investigation into the application of deep learning–based methods for the segmentation and voxel-level localization of MRONJ lesions on CBCT images. The proposed nnU-Net v2–based model demonstrated the ability to approximate localization of MRONJ lesions, while segmentation accuracy varied depending on lesion morphology and boundary definition. Given the limited sample size and the absence of external validation, the findings should be interpreted as exploratory rather than definitive clinical validation. Future studies incorporating larger, multi-center datasets and independent external testing are required to improve robustness and generalizability. Within these limitations, nnU-Net v2–based segmentation may serve as a supportive tool for radiographic assessment of MRONJ lesions rather than a standalone diagnostic or screening system. 

## Data Availability

The datasets used and/or analyzed during the current study are available from the corresponding author on reasonable request.

## Abbreviations

AI: Artificial Intelligence
AUC: Area Under the Curve
CBCT: Cone-Beam Computed Tomography
CLAIM: Checklist for Artificial Intelligence in Medical Imaging
CNN: Convolutional Neural Network
CT: Computed Tomography
DC: Dice Coefficient
DL: Deep Learning
DICOM: Digital Imaging and Communications in Medicine
FOV: Field of View
FP: False Positive
FN: False Negative
95% HD: 95% Hausdorff Distance
IoU: Intersection over Union
ML: Machine Learning
MRI: Magnetic Resonance Imaging
MRONJ: Medication-Related Osteonecrosis of the Jaw
NIfTI: Neuroimaging Informatics Technology Initiative
ROC: Receiver Operating Characteristic
STROBE: Strengthening the Reporting of Observational Studies in Epidemiology
TP: True Positive
